# New Aspects of Gene-Silencing for the Treatment of Cardiovascular Diseases

**DOI:** 10.3390/ph6070881

**Published:** 2013-07-19

**Authors:** Olivia Koenig, Tobias Walker, Nadja Perle, Almuth Zech, Bernd Neumann, Christian Schlensak, Hans-Peter Wendel, Andrea Nolte

**Affiliations:** Clinical Research Laboratory, Dept. of Thoracic, Cardiac and Vascular Surgery, University Hospital Tuebingen, Calwerstr. 7/1, 72076 Tuebingen, Germany

**Keywords:** RNAi, siRNA, ASO, transfection tools, delivery systems, atherosclerosis, CVD (cardiovascular disease), restenosis, drug eluting stents

## Abstract

Coronary heart disease (CHD), mainly caused by atherosclerosis, represents the single leading cause of death in industrialized countries. Besides the classical interventional therapies new applications for treatment of vascular wall pathologies are appearing on the horizon. RNA interference (RNAi) represents a novel therapeutic strategy due to sequence-specific gene-silencing through the use of small interfering RNA (siRNA). The modulation of gene expression by short RNAs provides a powerful tool to theoretically silence any disease-related or disease-promoting gene of interest. In this review we outline the RNAi mechanisms, the currently used delivery systems and their possible applications to the cardiovascular system. Especially, the optimization of the targeting and transfection procedures could enhance the efficiency of siRNA delivery drastically and might open the way to clinical applicability. The new findings of the last years may show the techniques to new innovative therapies and could probably play an important role in treating CHD in the future.

## 1. Introduction

Cardiovascular diseases (CVDs) are today, with approximately 17 million cases of death each year, the most common cause of mortality worldwide [1]. Because experts and the WHO expect an increase in cases of illness and deaths in subsequent years, there is the need for successful treatment of CVDs like atherosclerosis, hypertension, peripheral vascular disorders, *etc*. However, the ultimate goal to prevent pathogenesis of CVDs is to treat diseases which are responsible for this purpose, for instance hyperlipidemia, diabetes and hypertension in atherosclerosis. Currently, the possibilities are to prevent the progression of the diseases, an insufficient and unsatisfactory state. With growing knowledge about pathogenesis and its mechanisms, many promising approaches and efforts have been made in the field of gene silencing. RNAi was first described by Fire *et al*. in 1998 when they discovered gene silencing in *C. elegans* by double-stranded RNA [2]. It’s a powerful self-defense mechanism in *Eukarya* preventing infections and following genome integrity by viruses and transposons or can also regulate gene expression. In the last decade, this biological strategy has gained in importance and was used to examine the function of a gene of interest, to silence genes with pathological background or to knockdown foreign nucleic acids from virus or bacterial infections. Nowadays the role of siRNA and micro RNA (miRNA) as a potential therapeutic treatment in human gains center stage in RNA biology. *In vivo* gene knockdown studies are rising to prove their applicability in diseases like cancer, different kinds of infections or genetic disorders. Despite promising therapeutic possibilities, the current challenging aspects in RNAi mediated therapy are to improve the stability, the cellular uptake and the specific delivery of the siRNA focusing in this review.

Another method for interfering in transcription of genes is the antisense oligonucleotide (ASO) method, which is tested for cancer therapy, viral infections, autoimmune diseases and also CVDs. The three kinds of nucleotides for silencing genes have the same purpose: intervening in gene expression and preventing mRNA transcription by complementary sequences to target mRNA. However the mechanism of each method is different.

## 2. The Mechanism of RNAi and Antisense

**Figure 1:**
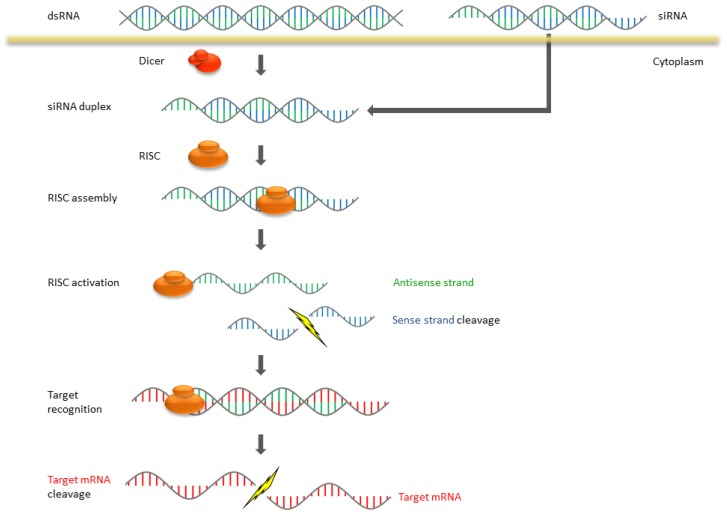
Mechanism of RNA interference (RNAi) in mammalian systems.

The expression of genes can be affected by siRNA capturing and finally cleaving complementary mRNA. RNAi is a multistep pathway which can be divided in two different phases: the initiation phase and the effector phase. SiRNA duplexes regulatory molecules with about 21–23 nt are processed in the initiation step by cleaving long dsRNA by RNase III which is called Dicer [3,4]. The cleaved siRNA contains a 5′-phosphate and a 3′-hydroxy group as well as 2 nt 3′-overhangs [5]. In the subsequent effector phase the siRNA is incorporated into a nuclease-containing RNA induced silencing complex (RISC) [6]. Within the complex an RNA helicase unwinds the siRNA and the RISC becomes activated. The single-stranded siRNA binds with the activated RISC at the target mRNA and leads to its degradation (Figure 1). While the RISC can be recovered for further cleavage cycles, the translation and expression of target mRNA are stopped.

In comparison to siRNAs, ASOs are single-stranded oligonucleotides with a variable number of nucleotides ranging from 13 to 25 [7]. Three different pathways are known to prohibit mRNA transcription like destabilizing the pre-mRNA, steric blocking of ribosomes and the most popular one which is already used in drugs: activating ubiquitous RNase H1 [7,8]. The enzyme provokes a hydrolysis of the RNA strand from a RNA/DNA complex and consequently blocks mRNA transcription.

## 3. Stabilization of siRNA and Its Delivery Agents

To overcome the degradation of free and naked siRNA *in vivo* and *in vitro* there is the need for stabilizing the nucleic acid. The endo- and exonucleases which are present in blood, serum, and in living cells provoke minimize the half-live [9,10]. Many efforts are taking place in the chemical sector in the last years to protect the siRNA from nuclease degradation. Maintaining gene-silencing activity is an important issue when modifying synthetic siRNA. Soutschek and his colleagues succeeded in improving the stability of their synthesized apoB-1-siRNA. They placed the focus on the backbone of the RNA and used phosphorothioate to stabilize the 3′ end of the sense and antisense strands. Furthermore, two 2′-*O*-methyl nucleotides were added at the 3′ end of the antisense strand and the sense strand was modified with cholesterol. Only the stabilized chol-apoB-1-siRNA was observed in the biodistribution in the liver and jejunum and showed reduced apoB mRNA levels, in contrast to the unconjugated apoB-1 siRNA [10]. The phosphorothioate and the 2′-*O*-methyl RNA modification induce resistance against nuclease digestion.

Another way to improve the stability and transfection rate was the use of a peptide transduction domain-double stranded RNA binding domain fusion protein (PTD-DRBD). Eguchi *et al*. revealed in their study that cationic PTDs neutralize the negative charge of nucleic acids by specific binding on the backbone of the dsRNA. The higher RNAi response after transfection, a slightly alteration in cell viability, and no cytotoxicity effects makes this construct a potent carrier for the transfection of primary cell types [11].

The negative charge of RNA enables the complexation with cationic polymers by electrostatic interactions. Nowadays, many different polymers used in several studies like peptides or proteins, natural and synthetic polymers are popular. The following sections give an overview of frequently used transfection reagents.

### 3.1. Liposomes and Lipids

**Figure 2:**
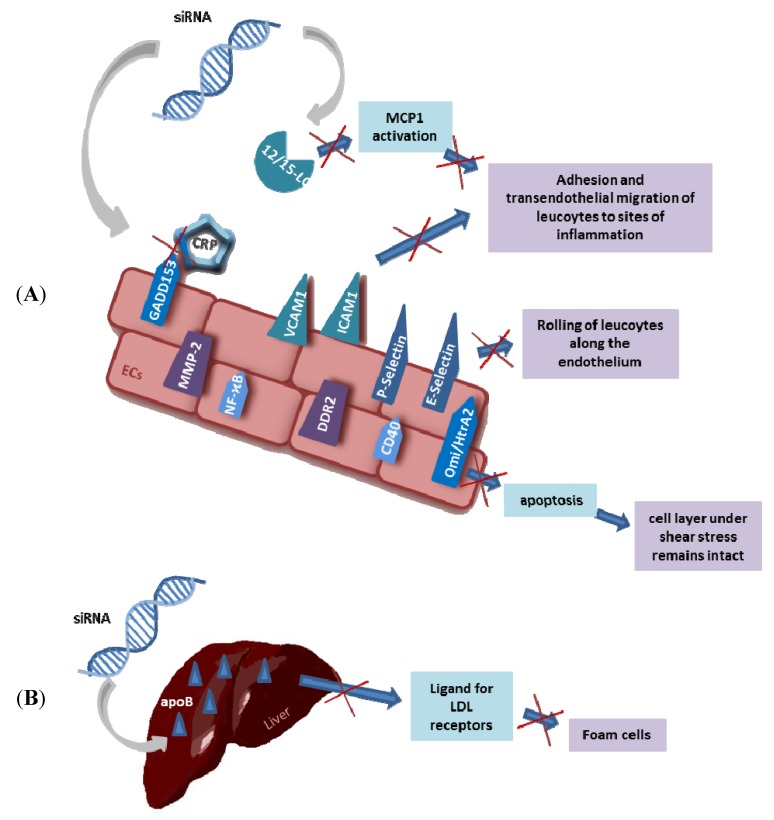
Potential targets and therapies for atherosclerosis with siRNA: (**A**) Several receptors are expressed by the endothelial layer which can be knocked down by siRNA and thus atherosclerosis can be reduced. (**B**) The liver expresses apoB (alipoprotein B). If apoB is knocked down by siRNA it does not act anymore as ligand for LDL receptors and therefore formation of foam cells is down regulated.

Liposomes or lipids are transfection reagents which are often used for *in vitro* and *in vivo* siRNA-transfection. Nucleic acids can be encapsulated as polar drugs in a bilayer of phospholipids improving the uptake of therapeutic siRNA by mediating endocytosis. The advantage of liposomes is that they can be produced as neutral or cationic liposomes with negative charged nucleic acid in the core. The charge of liposomes plays a key role in tissue distribution. Neutral liposomes are preferred for *in vivo* transfection and often used in tumor biology. Therefore, siRNA is incorporated into neutral 1,2-dioleoyl-*sn*-glycero-3-phosphatidylcholine (DOPC) liposomes and used efficiently in silencing genes involved in cancer. In an orthotopic mouse model of ovarian cancer the oncogene EphA2 could be silenced after three weeks of treatment with EphA2-targeting siRNA-DOPC and the tumor growth was reduced [12]. Halder and colleagues observed similar results by using focal adhesion kinase (FAK) siRNA-DOPC for ovarian carcinoma therapy. The mean tumor weight was reduced and the addition of a chemotherapeutic agent called docetaxel provoked a greater reduction in tumor weight [13]. These results were affirmed by the study about liposome-incorporated protease-activated receptor-1 (PAR-1) siRNA for targeting melanoma growth and metastases [14]. Neutral liposomes show generally good compatibility in *in vivo* application with low toxicity effect, whereas cationic liposomes which were used frequently in *in vitro* studies revealed some negative aspects. Because of the positive charge they can interact for instance with serum proteins, lipoproteins or with components of the extra-cellular matrix (ECM). The following consequences are aggregation, less stability of the complex or binding to unspecific cells [15]. Nevertheless, cationic lipids are often used in studies and like Oligofectamine (Invitrogen), Lipofectamine (Invitrogen) and RNAifect (Qiagen) are commercially available. Frequently, stable nucleic acid lipid particles (SNALP) are applied as liposomes for siRNA delivery. The advantages of these lipid nanoparticles are a high encapsulation rate of siRNA and small homogenous particle size. Zimmermann *et al*. were the first who showed gene silencing in non-human primates mediated by SNALP. The apoB-specific siRNA was encapsulated in nanoparticle lipids and intravenous injected in cynomolgus monkeys. The gene silencing provoked reduction in the apoB-100 protein levels and the cholesterol levels in according to the injected dose. As a consequence the LDL was reduced over the 11-day study and no complement activation, pro-inflammatory cytokine production or toxicities have been observed [16]. In matters of treating coronary artery diseases which are related with apoB and low-density-lipoprotein (LDL) levels this way of transfection reveals high potency (Figure 2B). Furthermore, combinations of SNALPs and siRNAs are successfully applied in studies for gene-silencing in tumors and viral infections [17,18,19,20].

### 3.2. Polyethylenimine: A Cationic Synthetic Polymer

Polyethylenimine (PEI) is the most popular synthetic polymer used for nucleic acid delivery. With its high cationic charge density it is able to non-covalently complex with the negative charged siRNA. As a result the nucleic acids are protected from digestion by different enzymes and endocytosis can take place. The polymer possesses an amino nitrogen on every third atom and is hence protonatable in an endosome [21]. PEI is able to buffer the pH in this system and as a result chloride anions flow in. This causes an influx of water into the endosome and osmotic swelling can occur. As a consequence of this “proton-sponge-effect” the endosome bursts and the PEI-siRNA complexes are released into the cytoplasm [22]. Two forms of the polymer, linear and branched, are common and available in different molecular weights. Low molecular-weight PEI is preferred in comparison to high molecular PEI due to the observed toxicity. The strong positive charge causes interactions with cells and their surface is destroyed. Zintchenko *et al*. improved the compatibility of branched PEI 25 kDa by modifying the polymer with organic substances like ethyl acrylate, succinic or acetic anhydride and 3-propionic acid. The toxicity could be decreased with all these modifications, whereas the neutral modified PEIs (ethyl acrylate and acetic anhydride) produced less cell viability than the PEIs with negative modification. Furthermore, the knockdown efficiency was higher after modification in comparison with the unmodified polymer. The modification with succinic anhydride and a modification degree of about 20% showed the most effective siRNA-mediated knockdown, even at low concentrations [23]. Given the fact that PEI (linear PEI 22 kDa and branched PEI 25 kDa) is not suited for siRNA delivery [24], different modifications enable a specific gene-knockdown by reducing the toxicity and stabilizing the polyplex. Another type of PEI is the linear 22 kDa jetPEI^©^ which is efficiently used for transfection of cell lines and primary cells. Urban-Klein *et al*. tested the cellular uptake and bioactivity of this transfection reagent in a stable luciferase expressing SKOV-3 ovarian carcinoma cell line. They noticed a dose-dependent reduction of the luciferase activity and proved that siRNA was protected against nucleases, that cells were transfected and that a gene-knockdown occurred. In their tumor xenograft mouse model they examined the efficacy of gene-knockdown of proto-oncogene HER-2 by systemic delivery of jetPEI^©^-siRNA. The naked siRNA was not present in the tissue and there was no change and reduction of tumor growth, respectively. However the complexed siRNA with jetPEI^©^ improved the efficiency of siRNA specific knockdown and thus reduced the tumor growth [25]. In another xenograft tumor model jetPEI^©^ was also successfully used for silencing FOXM1 which is overexpressed in human tumors. The jetPEI^©^-siRNA complexes which were directly injected into the tumor had a retention time of about 24 h and were therefore interesting for tumor therapy. Furthermore, this formulation was able to suppress FOXM1 reducing the mRNA levels [26]. When transfecting cells with PEI the ratio between PEI and nucleic acids, called N/P ratio (N = positive charged amine groups, P = negative charged phosphate groups), plays a pivotal role, besides the molecular weight and the chemical structure. It is known that the higher the N/P ratio of a PEI complex the smaller the size. For efficient gene delivery of these complexes into cells a size of about 200 nm should not be exceeded [27]. This fact is seen in the study of Wang *et al*. where they tested the luciferase silencing efficiency with different N/P ratios. The PEI-siRNA complexes with ratios 4 and 6 showed sizes above 200 nm and demonstrated half as much silencing efficiency in comparison to the complexes with higher ratios [28]. This result was confirmed in further studies by silencing VEGF gene with siRNA/PEI-PEG-PCP polyplexes [29] and by GFP downregulation with DOPE-PEI complexes [30]. The best N/P ratio has to be determined for every complex design and material. Several different studies in comparison showed a wide range relative to the ratio. Höbel *et al*. preferred a N/P ratio of 77 when transfecting cells with PEI F25-LMW (F = fraction, LMW = low molecular weight), which is a fraction of PEI 25 kDa with high molecular weight [31]. Lower ratios were used with *in vivo*-jetPEI^©^ (N/P = 6) [32] and multi-siRNA/PEI (N/P = 10) [33].

### 3.3. Atelocollagen: Collagen without Telopeptides

Atelocollagen is a natural polymer with low toxicity and low immunogenicity which is nowadays often used for gene delivery systems due to its biocompatibility. This desirable property is caused by pepsin treatment of collagen type I from calf dermis [34,35] and the high subsequent purification. The enzyme treatment provokes the detachment of the telopeptides (amino acid sequences) at the N- and the C-terminal ends of collagen which are responsible for the immunogenicity [36]. Atelocollagen is a potent transfection reagent for *in vivo* delivery of siRNAs owing to the absence of immunostimulation effects. While DOTAP and *in vivo* jetPEI induced IFN-α and -β release in immune stimulatory RNA (isRNA) intravenously injected Balb/c mice, complexed and uncomplexed atelocollagen provoked no stimulation of these inflammatory cytokines [37]. Atelocollagen is often used in clinical applications, for instance in wound-healing and as implants, where a biodegradable material is desired [38]. The advantage of this polymer is its availability in different states of aggregation when the temperature differs. At low temperatures (less than 10 °C) atelocollagen is liquid, whereas a gel is formed at 37 °C, provided that the concentration is above 0.5%. However, when the concentration is decreased, the polymer is similar in viscosity to blood. A study of Mu *et al*. used these facts for systemic and local delivery of siRNA with atelocollagen as transfection reagent. In a xenograft mouse model they confirmed the silencing of Bcl-xL, an anti-apoptotic protein, injecting the siRNA/atelocollagen complex into the tumor and intravenous, respectively. Furthermore, no intact Bcl-xL siRNA was seen in different organs such as brain, liver and spleen after systemic delivery, which argues for a high selective delivery system [39]. In a further xenograft model related to prostate cancer, siRNA/atelocollagen was used to silence the growth factor midkine (MK). Injecting the complex into the tumor, the expression and the secretion of MK were reduced. In combination with paclitaxel, a chemotherapeutic, the antitumorigenic effect of the MK siRNA could be amplified [40]. Noticing that neovascularization in tumors plays a decisive role in tumor growth, Takei *et al*. investigate in their study the silencing of VEGF *in vitro* and *in vivo*. The VEGF siRNA/atelocollagen combination reduced the tumor growth, and the microvessel density in the tumor was markedly lower in comparison to the tumors transfected with scrambled (unspecific) siRNA. The stability analysis of atelocollagen confirmed the protection of the siRNA injected in tumors. While VEGF siRNA/atelocollagen remained for at least 8 days in the tumor tissue, naked siRNA already showed low persistence at the first day, and at day 8 siRNA could not be detected [41]. Furthermore, a study of systemic delivery (by intravenous injection) of enhancer of zeste homolog 2 (EZH2) and phosphoinositide 3′-hydroxykinase p110-α-subunit (p110-α) siRNA/atelocollagen to bone-metastatic tumors revealed that atelocollagen can efficiently deliver nucleic acids to tumors and causes an inhibition of metastatic tumor growth. The experiment, using luciferase siRNA/atelocollagen, showed additionally that this complex was located in tissues like liver, lung, spleen and kidney with much higher amounts available than siRNA alone and that the retention time was longer [42]. Atelocollagen- mediated transfection also shows great potential for the treatment of muscular atrophy, a disease which still cannot be treated effectively. The studies of Kinouchi *et al*. demonstrated muscle growth *in vivo* by local and systemic delivery of siRNA targeting myostatin. Nanoparticles were formed with GDF8 siRNA26 (targeting myostatin) and atelocollagen and afterwards injected into the masseter and biceps femoris muscles. The sizes of the different muscle types were increased and even the myofibril sizes of the masseter were enlarged [43]. These results must be due to the diminished expression of myostatin by gene silencing. The caveolin-3-deficient mouse model of limb-girdle muscular dystrophy 1C (LGMD1C) confirmed such effects. The intravenous injection of myostatin siRNA/atelocollagen provoked a gain in several skeletal muscles and muscle fiber size. As a consequence the muscle fibers showed improved contractile force generation [44].

### 3.4. Chitosan: A Chitin Derivative

Chitosan is a natural, linear polysaccharide which consists of β-(1-4)-linked *N*-acetyl-D-glucosamine and D-glucosamine. Due to the low occurrence of this polymer it is generally produced by deacetylation of chitin, which is found, for instance, in crustaceans, fungi and insects [45,46]. If the *N*-acetylation of chitin is below 50%, the polymer is called chitosan, and it is soluble in acidic solutions [47]. In addition, the procedure (homogenous or heterogeneous conditions) used to prepare chitosan is a crucial factor for its solubility [48]. With its high positive charge, its biodegradability and a remarkable biocompatibility, chitosan is often used for siRNA delivery *in vivo* and *in vitro* [49,50]. A positive property of chitosan is that after enzymatic degradation the products are not toxic to organisms. For an efficient gene silencing, the pH value plays a pivotal role for building a complex between chitosan and the nucleic acid. Particles are formed if the p*K*_a_ value of chitosan is below 6.2–7.0 and consequently the primary amines of glucosamine are protonated and possess a positive charge [51]. Under this chemical condition, electrostatic interactions are formed by the positively charged ammoniums and the negatively charged phosphates of the nucleic acids. Furthermore, the strength of the interaction between chitosan and siRNA is higher in acidic solutions than under neutral or alkaline conditions. The high charge of chitosan is caused by the fully protonated amine groups and this leads to a suitable conformation for the interaction with nucleic acids. If the pH approaches the neutral range, the molecules of chitosan are unable to undergo extensive conformation transitions and hence the electrostatic contact groups are not available for interactions [52]. This aspect should be considered when forming stable nanoparticles with nucleic acids and biopolymers. In addition, the molecular weight, the degree of acetylation and the N/P ratio influence the complex formation [53,54]. Mittnacht *et al*. reported that their chitosan/siRNA nanoparticles are capable of inducing neurite outgrowth by silencing RhoA, which avoids the regeneration of a damaged nervous system, even when the inhibitory protein myelin is present. They further noticed that the particle size and the hydrodynamic diameter of the complex formed depends on the molecular weight of the chitosan, but is not relevant for the knockdown efficiency in their case. All formed particles had a hydrodynamic diameter below 350 nm and seemed to be good for cellular uptake [54]. The charge of the particles seemed to depend on the N/P ratio. SiRNA/chitosan complexes with an N/P ratio below 2 have a negative zeta potential and therefore a negative surface charge. Ratios above 2 provoke positive values and are consequently suited for a faster cellular uptake of nanoparticles [55,56]. This is maybe due to the electrostatic interactions which are built between the negative charge of the cell membrane and the positive charge of the nanoparticle [57]. SiRNA/chitosan complexes are often used to silence genes which are involved in tumorgenesis. In a xenograft nude mouse model of prostate cancer, nanoparticles of chitosan and siRNA were used for intratumoral injections. The relaxin family peptide receptor 1 was silenced and this resulted in a decreased tumor size, cell proliferation and increased apoptosis. The receptor and the peptide hormone relaxin have a pivotal role in several cancers, particularly prostate cancer. The tumor seems to be more aggressive if the concentration of relaxin is increased [58]. The study results are promising for a prostate cancer therapy. Similar conclusions were reached by Ji *et al*. and Lu *et al*. with chitosan nanoparticles for silencing genes like the oncogene four and a half LIM domains protein 2 (FHL2) and EZH2 [59,60]. Furthermore, siRNA/chitosan nanoparticles are often combined with polymers for delivery into cells. A quite new study examined for the first time the release mechanism of PLGA nanofiber-encapsulated siRNA/chitosan nanoparticles. It is already known that encapsulation of drugs leads to enhanced drug efficiency, specificity and controlled release [61]. PLGA encapsulation seems to protect the siRNA/chitosan complex from degradation under basic conditions and prolongs its bioactivity [62]. Besides the nanoparticle delivery system, chitosan hydrogels were recently used for local gene silencing. Due to its temperature-dependent aggregate state, chitosan can be used as a liquid siRNA complex, which could be injected into mice in a tumor xenograft model. After intratumoral injection, the liquid chitosan forms a hydrogel and the release of siRNA could be proved. The TG2-targeted siRNA hydrogel caused reduction in tumor volume in comparison to scramble siRNA. This effect could be reinforced by adding docetaxel, which was added to the siRNA/chitosan solution before the injection [63].

### 3.5. Hyaluronic Acid: Component of the ECM

Hyaluronic acid (HA) belongs to the negatively charged glycosaminoglycans and consists of D-glucuronic acid and *N*-acetyl-D-glucosamine linked by β-1,4- and β-1,3-glycosidic bonds [64]. The polysaccharide occurs naturally and is the major component of the ECM. Furthermore, it is located in biological fluids like synovial fluid, umbilical cord and blood with different molecular weights ranging from 10^3^–10^7^ Da [64,65,66,67]. HA possesses the ability to accumulate water in large amounts and therefore has a broad viscosity range. The biopolymer is further characterized by biocompatibility, biodegradability and no signs of immunogenicity which makes it optimal for use in wound healing, in the treatment of knee osteoarthritis and for tissue-engineered scaffolds [68,69,70,71]. For the extraction of HA, rooster combs or human umbilical cords were used primarily [72,73]. However, the extraction and purification of HA from these sources is complex, resulting in high end costs [74]. Furthermore, the clinical application of animal HA is critical due to the possibility of contamination with viral agents. Nowadays a fermentation process mainly suing *Streptococcus zooepidemicus* is the major industrial source of HA. Non-pathogenic mutants are used and there is obviously no batch-to-batch variation and the encapsulated HA can be easily extracted [75]. Nevertheless, *Streptococcus* sp. have the ability to produce endotoxins and therefore, recombinant production of HA with Gram-negative and -positive bacteria comes to the fore [76,77,78]. HA is not a typical transfection reagent because of its anionic charge, but it is often used in combination with other biomaterials like chitosan, poly-L-arginine, collagen or the polymer PEI for nucleic acid delivery and it possesses the ability to reduce the cytotoxic effects of PEI used in transfection reactions [79,80,81,82,83]. In addition, the ternary complex siRNA/HA-PEI provokes a higher gene knockdown in comparison to PEI combined with siRNA, which was proved by Han *et al*. with different tumor cell lines [82]. A more effective ternary silencing complex has been achieved using redox-responsive hyperbranched poly(amidoamine) (PCD) instead of PEI in combination with siRNA and HA. Comparing PCD ternary complexes with PEI ternary complexes, the PCD ones provoked a higher reduction in VEGF expression in breast cancer cells. It is thought that PCD could better release the siRNA than PEI. Furthermore, the mixing order of the combination siRNA/PCD/HA influences the efficiency of the transfection and the gene knockdown [84]. Applying siRNA/HA complexes in the treatment of tumor diseases, receptor mediated endocytosis could be the moving force of effective gene silencing. The cell-surface receptor CD44 is frequently overexpressed in different kinds of tumors in comparison to the respective normal tissue, and is therefore an excellent target for cancer treatment [85]. HA interacts with further cell surface receptors like hyaluronan-mediated motility receptor (RHAMM) and intracellular adhesion molecule 1 (ICAM-1) which provoke different cellular processes, for instance migration, proliferation, differentiation, and angiogenesis [86,87,88,89,90]. Using HA for gene silencing complexes, nanoparticles are assembled and applied in many studies due to the possibility of simple application. HA coated lipid-based nanoparticles (LNPs) were tested for delivering siRNA into cancer cells. Only HA grafted LNPs were internalized into human lung adenocarcinoma A549 cells and siRNA against the p-glycoprotein induced a decrease in the mRNA and protein level in the human ovarian cell line, NCI-ADR/Res. The peculiarity of these HA-LNPs is that there is no cytotoxicity and no release of immunogenic reactions, an advantage compared to cationic molecules applied in transfections [91]. To reinforce the decrease of cytotoxicity, Raviña *et al*. formed chitosan nanoparticles combined with PEG and HA, substances which favor that. The cell viability could be improved by increasing the HA content in the HA/chitosan-g-PEG nanoparticles, when the nanoparticle dose for transfection was augmented. The gene knockdown of Snail1, a transcription factor which is involved in tumor invasive, was achieved and reached values comparable with Lipofectamine 2000 [80]. Al-Qadi *et al*. reported correlating viability and knockdown results comparing chitosan and HA/chitosan nanoparticles. The luciferase expression in A549 cells could be diminished to the level of Lipofectamine™ RNAiMAX only when HA is included in chitosan nanoparticles with an increasing ratio [83].

## 4. Delivery Systems

**Figure 3:**
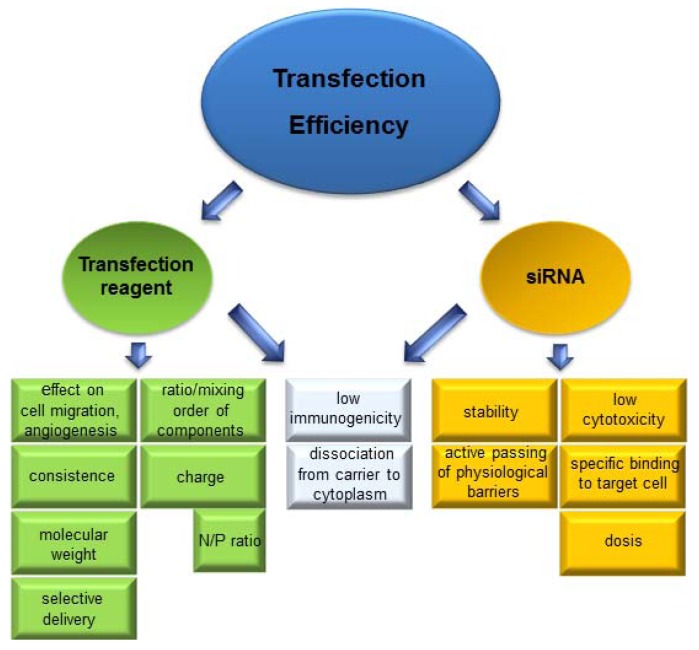
Important factors for siRNA delivery. Several parameters of transfection reagent and siRNA should be considered to gain high transfection efficiency. Because of no passive diffusion of siRNA there is the necessity for a transfection reagent which enables penetration through the cell membrane and release of siRNA for gene silencing.

The pivotal point of RNAi feasibility in *in vivo* and *in vitro* applications is the transfer of siRNA into the cytoplasm and a high transfection rate of the target cells, respectively. The molecular characteristics of siRNA hinder the entrance of naked siRNA through the cell membrane which is necessary for interactions required in the transcription process. The high polyanionic charge with about 40 negative phosphate charges cannot pass the hydrophobic cell membrane by passive diffusion, and also the molecular weight of about 14 kDa prohibits it [92,93]. In addition, the rapid enzymatic degradation of siRNA due to the low stability of the nucleic acids calls for a delivery system which facilitates the transfection of target cells and the following sequence-specific gene knockdown. An overview of critical parameters for siRNA transfection is given in Figure 3.

For siRNA transfer into cells, viral vectors are clearly superior to non-virus vectors such as liposome siRNA, and polymer siRNA complexes. Viral vectors have a high transfection efficacy due to their specific surface receptors which allow binding, internalization and delivery of genetic material into cells. The most commonly used viral vector families are adenovirus, lentivirus and adeno-associated virus [94]. However, there are some limitations and disadvantages in using viral vectors, e.g., adenovirus can lead to a massive immune response and to destruction of transfected cells [95]. Other problems are neutralization of vector DNA by preexisting antibodies that reduces transfection efficiency and wide tissue distribution that reduces target selectivity [96]. After intramuscular, intrabronchial, hepatic artery and subretinal administration, low-levels of vector of adeno-associated virus (AAV) DNA were found in body fluids and distal organs during biodistribution studies in non-human primates. Even though no causal relationship has been established, besides cellular and humoral immune responses, a correlation between early abortion, male infertility and the presence of AAV DNA in genital tract is also assumed [97].

## 5. Systemic Delivery Systems

**Figure 4:**
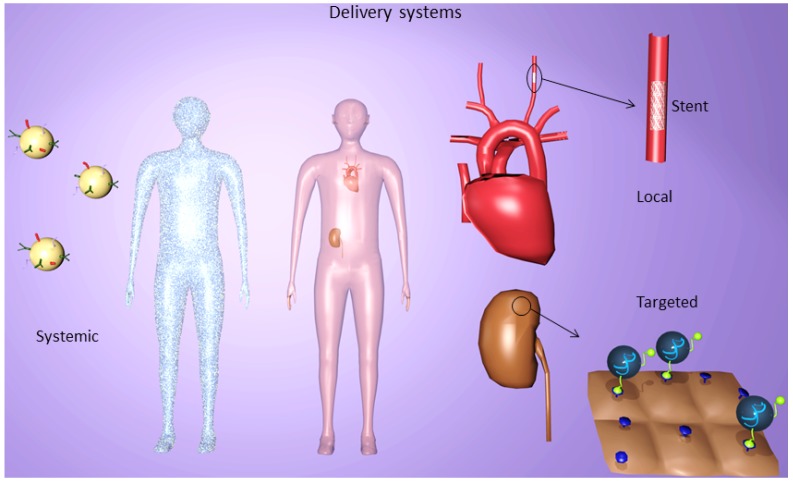
SiRNA delivery systems for *in vivo* applications. Systemic, targeted and local delivery of siRNA serve as approved systems in clinical therapeutics. Every application form possesses its own advantages and disadvantages due to their different characteristics of localization. Systemic administration provokes a completely distribution of drugs in the organism, whereas a specific release of siRNA can be reached by targeted and local delivery using for instance stents or modified NP.

Gene silencing by systemic delivery is a model which is easy to realize in clinical applications. It is often utilized in therapeutic cases, when the target of interest is quite difficult to access or widely spread throughout the body like in cases of metastases. Systemic delivery of small interfering RNA as cancer therapy might be the field of greatest interest in research. In the last few years mainly targeted delivery systems which can bind specifically to tumor cells have been developed [98,99,100]. The systemic administration of these drugs occurs by intravenous or intraperitoneal injections and sometimes by oral intake of medication [101,102,103,104]. However, this delivery system is not tissue specific (Figure 4) and therefore one must be aware that the gene-knockdown should not be able to influence the function of other cells and tissues which are not involved in the disease. Shi *et al*. examined the biodistribution and kinetics of siRNA delivered by LNPs after tail vain injection in a mouse model. They studied the delivery of siRNA against the Sjogren syndrome antigen B (Ssb), a ubiquitously expressed antigen, by using immunofluorescence (IF) staining and quantitative polymerase chain reaction (qPCR). 0.5, 2, 6, and 24 h post dosing different organs were collected and the levels of siRNA were measured. The ranking after 0.5, and 2 hr was liver > spleen > kidney > lung > heart, and low levels in brain and duodenum. Corresponding to that the Ssb mRNA knockdown after 24 h was about 85% in the liver and 25% in the spleen. In the other organs no significant knockdown could be seen [105].

Despite substantial progress in siRNA delivery to liver tissue [106], efficient systemic siRNA delivery to extra-hepatic cells in tissues is still a hurdle to be overcome. Novobrantseva *et al*. developed a systemic delivery method for RNAi-mediated silencing, especially in myeloid cells. They tested two diverse formulations of lipid nanoparticles encapsulating siRNA directed to various myeloid-expressed gene targets (CD45, CD11b, integrin β1, TNFα) in mice. Robust delivery to the spleen could be shown, whereas the peaking fluorescence signals in liver, gall bladder and intestine seemed to reflect the mode of excretion. The highest knockdown of mRNA levels 3 days after a single i.v. bolus injection (80%) has been seen in cells with a macrophage lineage. Dendritic cells showed 40% knockdown and B-cells about 15%. Silencing in all cell types was tested in different tissues including blood, spleen, bone marrow, liver and the peritoneal cavity. Unexpectedly the strongest silencing of CD45 in mice, with up to 90% reduction, was seen in the peritoneal cavity, whereas the myeloid cells in the peritoneal cavity of nonhuman primates treated with the same protocol did not show any detectable knockdown [107]. Leuschner *et al*. were able to silence chemokine (C-C motif) receptor 2 (CCR2), which is responsible for the distribution of inflammatory monocytes to the site of inflammation, leading to efficient reduction of inflammation in different disease models. By dynamic tomography they could see that the fluorescent tagged siRNA distributed from the blood stream to the spleen and bone marrow. By targeting the recruitment mechanism of inflammatory monocytes, the innate immunity remains largely unaffected [108].

Santel *et al*. developed a cationic lipid-based formulation of siRNA that improves the biodistribution properties in terms of enhanced uptake of siRNAs into endothelial cells in mice. SiRNA-Cy3-lipoplexes accumulated especially in the spleen and liver. However, the higher siRNA uptake of an organ does not automatically indicate that the siRNA entered the cytoplasm of the cell, what is albeit a precondition for RNA interference. By confocal microscopy they detected that the fluorescence staining was mainly present in the linings of the blood vessels. Counterstaining with an anti-CD31 antibody assured these findings. Furthermore, they analyzed the mRNA knockdown of CD31 and Tie2 which are only expressed on endothelial cells by Real-Time-PCR (normalized to CD34, another gene with restricted expression on endothelial cells). Significant gene silencing was seen for both genes CD31 and Tie2 in lung, heart and liver. Unexpectedly, no suppression of endothelial gene expression could be observed in spleen and kidney, despite obvious siRNA-Cy3 distribution in these organs [109]. In another study they showed appropriate results for the predominant uptake of their cationic lipid-based siRNA formulations to the endothelial cells of liver and tumor blood vessels, which could be the basis for antiangiogenic cancer therapies [110].

Oral delivery of siRNA is a very rarely used and investigated as a way of administration. Extreme pH alterations in the gastrointestinal tract and inefficient absorption are just two of many hurdles that have to be overcome. Zhang *et al*. designed different ternary polymeric nanoparticles and used a TAMRA-labeled control siRNA for their *in vivo* trials in rats. They analyzed the plasma levels and tissue concentrations 2, 6, and 12 h after oral administration. A good systemic biodistribution could be observed, and especially in plasma, liver and spleen, high concentrations of siRNA could be seen 6 h post oral administration [111].

## 6. Targeted Delivery Systems

Targeted delivery of siRNA is an appropriate gene delivery strategy to prevent unspecific gene delivery and undesired side effects which can occur by systemic applications of siRNA. In clinical applications there is the need to accumulate the gene silencing therapeutic at the place of disease without causing silencing effects in uninvolved cells or tissues. Furthermore, the circulation time must be prolonged and there is the aim to reduce the amount of drug used for systemic delivery (Figure 4). Several studies exhibit different approaches for cell-surface receptor mediated siRNA incorporation by modifying the transfection complexes. Folate-mediated uptake of siRNA is an excellent strategy for gene silencing of many cancer cells (brain, kidney, ovary, breast, lung and myeloid cells) due to their overexpression of the folate receptor [112]. The high-binding affinity of about K_d_ = 10^−9^ M to the folate receptor guaranteed the uptake of folate complexes and their endocytosis [113]. Folate can be covalently linked to siRNA by a poly(ethylene glycol) (PEG) spacer which additionally stabilizes the nucleic acids. However, siRNA/folate complexes are not able to silence gene expression due to their entrapment in endosomes [114,115]. Successful gene knockdown could be gained when siRNA/folate-PEG complexes were modified with the monodisperse polycationic carrier 386 [115]. An *in vivo* study with BALB/c mice, in which Colon-26-luc cells (express stably luciferase gene) were injected, showed great therapeutic potency for the transfection complex folate-PEG-appended polyamidoamine dendrimer. The luciferase activity could be reduced to 40% in comparison to control applying the folate combined dendrimer. The silencing effect of the dendrimer without folate showed higher luciferase expression, meaning that folate ligands could reinforce the siRNA uptake in tumor cells. In addition, folate could mediate a cell or tissue specific gene silencing, when it was conjugated to liposomes to knockdown the proto-oncogene *MYCN*. A preliminary study with severe combined immune deficient (SCID) mice bearing a LA-*N*-5 xenografted tumor demonstrated a receptor-mediated uptake of the liposome-siMYCN folic acid complex and consequently 53.1% knockdown of mRNA expression in tumor tissue [116]. Liposomes or LPNs not only play a major role in systemic delivery but also gain in importance in targeted delivery systems due to the possibility of modifying surfaces. Another possible way to combat neuroblastoma with liposomes is to decorate them with antibodies against disialoganglioside (GD2), which is highly expressed in neuroblastoma cells. Two different kinds of anti-GD2-liposomes provoked an enhanced internalization of the siVEGF complexes in comparison to the unmodified ones and VEGF-A was only reduced by the antibody coupled liposomes [117]. However, the surface charge could additionally influence the uptake of these liposomes noticing a higher uptake by the positively charged ones. Targeting liver cells for the protection of fulminant hepatitis LNPs could be coupled with galactose (Gal), which binds to hepatocyte specific asialoglycoprotein receptor. The reduction of apoptosis could reached by Gal-LNPs containing Fas siRNA, a protein which is involved in apoptosis in hepatocytes. A significant Fas mRNA reduction in a concanavalin A induced hepatitis model was seen when treated with Gal-LNPs-Fas in comparison to the control groups without Gal, naked Fas and Gal-LNPs with Gl2 [118].

The arginine-glycine-aspartate (RGD) sequence is especially utilized for tumor targeted siRNA delivery with interest in the *α_ν_β_3_* integrin, a receptor overexpressing in tumoral endothelial cells and different kinds of tumors. The specifically and preferentially binding of RGD peptide to the *α_ν_β_3_* integrin receptor is the reason for great promising results. Han *et al*. covered siRNA/chitosan-Nanoparticles (NP) with RGD peptide. SKOV3ip1 cells, which express *α_ν_β_3_* integrin, showed a higher uptake of the NP when they were covered with the RGD peptide than without. *In vivo* examinations of SKOV3ip1 bearing mice provoked a 3-fold higher appearance of siRNA when the NP was coupled with RGD. Gene silencing of periostin (POSTN), responsible for cell survival, invasion and angiogenesis, reached higher values in siRNA/RGD-chitosan NP treated tumors than NP without RGD and additionally the tumor growth was more inhibited [119]. Recently, studies also demonstrated successfully constructed gene carrier with cyclic RGD (cRGD) sequences [98,120,121]. PEGylated polycation liposomes, containing siRNA for c-myc, MDM2, and VEGF, were coated with cRGD peptide. *In vivo* examination in B16F10-bearing mice showed the highest uptake of complexes in the lung with metastases and the highest suppression of metastatic tumor when they were additionally prepared with cRGD [98]. Antibody targeted delivery is a meaningful option in presence of overexpressed or solely expressed antigens on target cells. Due to expensive production of antibodies in mammalian cells, antibody fragments building in bacteria are cumulatively utilized. These fragments possess further advantages relating to cellular mechanisms compared to antibodies. If the antibody loses its Fc domain, it won’t aim to bind to non-target cells like macrophages, which possess Fc receptors. Furthermore, the smaller fragment size facilitates their uptake in tissues [122]. Song *et al*. demonstrated in their study the cell specific targeting by antibodies by applying a heavy chain Fab fragment which was fused to protamine and was directed to the envelope glycoprotein (gp120) of human immunodeficiency virus (HIV). Only envelope-positive cells could be transfected with the fusion protein and provoked gene silencing. The functionality of the fragments could be confirmed by testing a single-chain antibody fragment ML39 (scFv) which targets ErbB2 receptor. The suppression of target genes occurred only in ErbB2-positive cells [123]. Another scFv-protamine fusion protein was used in a study targeting Her2, a receptor which is overexpressed in breast cancer cells. Breast tumor xenograft-bearing BALB/c nude mice with either Her2^+^- or Her2^−^-receptor demonstrated different results. Only in the Her2^+^-positive xenografts and tumor blocks, respectively, could complexed FAM-siRNA be detected. Examination of Polo-like kinase 1 (PLK1) gene silencing effect of breast cancer cells indicated a specific gene knockdown in Her^+^-positive cells suggesting an increased apoptosis and reduced proliferation which leads to suppression of tumor growth [124]. Applying antibody-coated siRNA particles for gene silencing in comparison with commercial available transfection reagents, a similarly abatement of mRNA expression was seen. However, the cytotoxicity is significantly lower using antibody-siRNA particles with, for instance, chitosan [125].

## 7. Local Delivery Systems

Site-specific delivery targeting can be achieved by local applications to different kinds of organs or tissues like eyes, skin or lung. This kind of siRNA release avoids the undesired treatment of systemic off targets, reduces the amount of drugs necessary and enables site-specific release [126]. Nowadays some local delivery methods have already been successfully tested in clinical trials, like in wet-age macular degeneration (AMD). Here, new blood vessels are built behind the retina leading to a loss of function of the tissues around the *macula lutea* to the point of loss of vision. The intraocular delivery by injection of siRNA Bevasiranib (Acuity Pharmaceuticals), targeting VEGF, demonstrated great potential for inhibiting neovascularization as well as Ranibizumab (Merck-Sirna therapeutics) also known as Sirna-027/AGN211745 [127]. Respiratory diseases, induced by virus infections, seem predestined for local delivery of siRNA drugs due to the possibility of simple intranasal application. The anatomy of the respiratory tract enables the dispersion of drugs including the epithelial cells in the trachea up to the smallest cells, the alveoli [128]. An antiviral siRNA called ALN-RSV01 was established by Alnylam Pharmaceuticals for the treatment of the respiratory syncytial virus (RSV) with the target mRNA of its nucleocapsid protein. A significant antiviral effect was observed and the drug provoked no side effects compared with a saline placebo [129]. Local delivery of siRNA worked in a study by protecting mice from lethal herpes simplex virus 2 (HSV-2) infection as well. Only the vaginal and ectocervical epithelial cells internalized the siRNA combined with oligofectamine by intravaginal release and no distant tissues were transfected. Up to at least nine days the siRNA could be detected and protected mice both before and after a lethal HSV-2 infection [130]. A great promising result for inhibiting scars during the healing of full thickness skin defects was recently shown by Liu *et al*. A collagen-chitosan/silicone membrane bilayer dermal equivalent (BDE) served as a matrix connecting with siTGF-β1/trimethylchitosan complexes to intervene into its signal pathway and interrupt scarring. The porcine model with full-thickness wounded backs demonstrated the effectiveness of this gene silencing method. The expression of mRNA and protein level of TGF-ß1 could be reduced and as a consequence no nodularity, a flatter skin surface, less scarring and a dermal-like structure was determined in comparison to the BDE without siRNA [131]. Another siRNA-carrier consisting of an electrospun PCL-PEG nanofibrous mesh with incorporated siRNA was tested for the treatment of diabetic ulcers in normalizing the overexpression of MMP-2. The siRNA nanofibrous meshes depositing on burn wound female C57BL/6 mice with induced diabetes mellitus symptoms provoked at day 7 an extreme decrease of the MMP-2 expression. Furthermore, neo-collagen was found at the wound sites, an ECM protein which enables the cell infiltration and consequently the building of new tissues [132]. Tumor treatment by local delivery of an injectable, thermosensitive hydrogel exhibits an interesting starting point for long-term therapy and long-term release of siRNA, respectively. Polyplexes were constituted of low molecular weight PEI-poly (organophosphazene) and siRNA targeting cyclin B1, a protein which controls the cell cycle. The injection of the liquid into the target tissue of PC-3 transfected Balb/c nude mice provoked the phase transition into a solid and a located hydrogel was formed. After 30 days, the tumor growth could be successfully inhibited with a near disappearance of the tumor [133]. Systemic and local delivery of siRNA is also interesting in the field of atherosclerosis and associated restenosis. The application of systemic siRNA delivery and also local delivery by stents might be one method of choice in this disease.

## 8. Chances for Atherosclerosis Therapies

Atherosclerosis is an inflammatory process which represents the most common cause for cardiovascular diseases (CVD) like myocardial infarction or stroke [134]. In 2008 more than 17 million people died from CVD, representing 30% of all global deaths [135]. During the inflammatory process of atherosclerosis, (LDL) cholesterol causes accumulation of lipids within the artery wall [136,137] followed by several mechanisms like lesion initiation, plaque rupture and thrombotic vessel occlusion [1].

To combat atherosclerosis several receptors participating in the inflammatory process are in focus to be controlled by siRNA technology. The expression of cell adhesion molecules (CAMs) like ICAM-1, vascular cell adhesion molecule 1 (VCAM-1) by endothelium is an indicator for inflammation. Selectins like endothelial-leukocyte adhesion molecule (E-selectin or ESEL) and platelet-leukocyte adhesion molecule (P-selectin or PSEL) bind to carbohydrate ligands on leukocytes. It is assumed that ICAM-1, VCAM-1 and E-/P-selectin take part in the causal pathway leading to atherosclerosis as selectins mediate rolling of leukocytes along the endothelium and ICAM-1 and VCAM-1 lead to adhesion and transendothelial migration of leukocytes [138,139]. During development of atherosclerosis vascular smooth muscle cells (VSMCs) change their phenotype from the quiescent “contractile” phenotype to the active “synthetic” phenotype and migrate from the media to the intima of the arterial wall, where they generate ECM leading to the formation of intimal lesions [140]. Petersen *et al*. isolated smooth muscle cells (SMCs) from aorta of C57BL/6 mice and showed that after transfection with siRNA against VCAM-1, migration of cells in a scratched area of a monolayer was significantly reduced, compared to non-transfected cells [141]. Hence VCAM-1 affects migration of SMCs in the intima and knockdown of this adhesion molecule could be a promising tool for treatment of atherosclerosis. Qu *et al*. demonstrated that VCAM-1 plays an important role in the pathogenesis of neointimal hyperplasia in rat carotid artery. Restenosis after carotid surgical mechanical de-endothelialization (CSMDE) is reduced, if VCAM-1 expression is inhibited by siRNA, thus siRNA against VCAM-1 could be a therapeutic option against atherosclerosis as well [142]. Another possibility to stop inflammatory processes is to inhibit leukocyte entry to lesion areas. If CD40-CD154 signaling is interrupted by siRNA knockdown of CD40, expression of VCAM-1, ICAM-1 and E-selectin is inhibited and thus leucocyte adherence reduced [143]. Wang *et al.* also showed that silencing of CD40 by lentiviruses carrying siRNA leads to regression of atherosclerotic plaques in carotid arteries of apolipoprotein E-deficient (apoE−/−) mice [144]. An overview of possible targets and therapies for atherosclerosis is shown in Figure 2.

Although migration and accumulation of SMCs in the intima is a key event in the development of atherosclerosis, apoptosis of SMCs also plays an important role in development of atherosclerotic lesions and increased plaque vulnerability. C-reactive protein (CRP) circulates as a pentamer (pentameric CRP) in plasma and is seen as a predictor of coronary events [145]. Blaschke *et al*. demonstrated that CRP induces caspase-mediated apoptosis of coronary VSMCs. GADD153 is a gene involved in growth arrest and apoptosis in vascular and nonvascular cells. After transfection with siRNA against GADD153 gene CRP-induced apoptosis was significantly reduced [146].

MMP-2 is expressed in VSMCs. Increased expression leads to hydrolysation of numerous ECM components, which facilitates VSMC migration and fibrous cap degradation [147]. In apoE−/− mice Kuzuya *et al*. proved that MMP-2 deficiency reduced atherosclerotic plaque lesions and SMC accumulation in plaque lesions [148]. Hlawaty *et al*. tested the effects of MMP-2 siRNA on rabbit VSMCs *in vitro* and *ex vivo*. They could show that transfection of intimal cells with MMP-2 siRNA leads to inhibition of MMP-2 activity [149]. Discoidin domain receptor 2 (DDR2) takes part in regulation of collagen turnover, mediated by SMCs in development of atherosclerosis. After carotid injury level of DDR2 significantly rises, Shyu *et al*. discovered that siRNA mediated silencing of DDR2 down-regulates neointimal formation in balloon injured rat carotid artery [150].

The leukocyte-type 12/15-lipoxygenase (12/15-LO) enzyme plays an important role for mediating LDL oxidation and development of atherogenesis by promoting endothelial inflammation and foam cell formation [151,152,153]. The chemokine monocyte chemoattractant protein-1 (MCP-1) leads to migration of circulating leukocytes to sites of inflammation and its expression is linked to the activation of 12/15-LO [154]. Dwarakanath *et al*. reported decreased MCP-1 mRNA levels and NF-ϰB expression in 12/15-LO knockout mice. Furthermore, they designed siRNA to knockdown NF-ϰB p65 in human VSMCs and they observed 83% less MCP-1 expression after TNF-α stimulation compared to the cells which were transfected with the vector alone [155].

If the proapoptotic mitochondrial serine protease Omi/HtrA2 is overexpressed, cells are sensitized to apoptosis. SiRNA against Omi/HtrA2 reduces cell death [156]. Sun *et al*. exposed human umbilical venous endothelial cells (HUVECs) to 15 dyne/cm^2^ shear stress, which inhibited expression of Omi/HtrA2 at mRNA and protein levels. Furthermore, if fibroblast growth factor was removed from medium and FBS decreased, release of Omi/HtrA2 from mitochondria was induced. Shear stress inhibited its release under the same conditions. Hence, down-regulation of Omi/HtrA2 by siRNA may contribute to the positive effect of shear stress on endothelial cells by additionally stopping cells from entering apoptosis and thus prevent atherosclerosis [157].

## 9. Local Delivery of siRNA for Atherosclerosis and Restenosis Control

Nowadays drug eluting stents (DES) are well accepted for the treatment of arterial diseases and replace the conventional therapy with bare metal stents [158]. Good progress could be achieved to reduce the threat of restenosis by neointimal hyperplasia, and the re-endothelialization of the stent could be accomplished [159]. However, a suitable stent for post-DES without side effects has not yet been found. Gene silencing stents describe a new promising therapeutic strategy releasing specific siRNAs to the endothelial cells of vascular wall for its regeneration and inhibition of restenosis (Figure 4). In a preliminary *in vitro* study of intravascular local gene silencing, the expression of E-selectin (ESEL) could be down-regulated when endothelial cells from human saphenous veins were seeded onto a gelatin coating combined with transfection complexes which consist of siESEL/PEI [160]. In the field of bioactive coronary stents, polyelectrolyte multilayers (PEM) seem to have great potential for inhibiting restenosis. Hossfeld *et al*. established with the layer-by-layer technology multilayers consisting of chitosan and HA with included fluorescence labeled siRNA/chitosan NP. Due to the important point of long-term siRNA release in clinical application, the tests showed a decreased removal of siRNA NPs to ECs between days two and six. The coated stents were tested in an *ex vivo* model with carotid porcine arteries and provoked the uptake of siRNA/chitosan NPs into the artery walls. The good hemocompatibility of the multilayers and the possibility of sterilizing the bioactive stents with ETO make this system a potential candidate for combating coronary diseases [161]. Recently, San Juan *et al*. already developed a gene silencing stent coating as to a local therapy in the artery walls, covered with cationized pullulan hydrogel and loaded with siRNA targeting MMP2 in VSMCs. In the *in vivo* study with balloon-injured hypercholesterolemic carotid arteries of white rabbits the implanted siMMP2 loaded stent led to a significant down-regulation of pro-MMP2 and a decrease in MMP2 activity. The gene specific targeting of this stent could be proved as pro-MMP9 activity was not influenced [162]. Li *et al*. demonstrated a knockdown of NOX2 (*Cybb*) by a new approach using the amino-acid-based nanoparticle HB-OLD7 in a rat model. NOX2 is a functional component of the phagocyte NADPH oxidase and it seems to be the major oxidase affecting human intimal SMCs. After balloon angioplasty of carotid artery in an atherosclerotic rat model the HB-OLD7-siSTABLE-Cybb (siCybb) complex was delivered into the adventitia and *Cybb* gene and NOX2 protein expression as well as ROS production and neointima formation were analyzed. All showed significant reduction and no significant change of *Cybb* gene and NOX2 protein levels was observed in spleen. Thus local, arterial wall transfer with HB-OLD7 nanoparticles could be a promising method for nonviral and efficient knockdown of NOX2, which is a meaningful participant in development of restenosis and atherosclerosis [163]. Another study focused on Akt1 protein which has an important role in cellular proliferation. Che *et al*. formed siAkt1 NP combined with a disulfide cross-linked low molecular polyethylenimine (ssPEI) and deposited them onto HA-precoated stents. Preliminary studies with A10 VSMCs demonstrated the efficiency of this delivery system since the Akt1 mRNA and protein expression was reduced by releasing NP. Furthermore, the downstream signaling proteins, like mTOR, 4E-BP1, and p70S6K were down-regulated as well. In the *in vivo* model with New Zealand white rabbits, the in-stent restenosis (ISR) area of the implanted and after four weeks removed stent was calculated. The bare metal stent served as a control and showed an increasing ISR area in comparison to the siAkt1-PEI/HA coated stent. That confirmed the efficient Akt1 gene silencing effect, which lead to the suppression of overgrowing VSMCs and consequently to cell death [164].

## 10. Treatment of Atherosclerotic Risk Factors by siRNA and ASOs

### 10.1. Hypertension

Hypertension is often associated with atherosclerosis as it is known to be a high risk factor for its pathogenesis and further cardiovascular diseases like stroke, myocardial infarction, and aneurysms. Different possible targets to combat hypertension using siRNA technology could be figured out. Pharmacologic blockage of α1-adrenergic receptor (AR), located on VSMCs, is widely applied for the treatment of hypertension. Therefore, Sun *et al*. examined the silencing effect of siRNA targeting the mRNA of α1_D_-ARs in rats. The time-dependent decrease of the α1_D_-AR protein level raises high interest for further examinations [165]. Blocking receptors of the renin-angiotensin-aldosterone system (RAAS) could be another method for influencing the blood pressure (BP). Aldosterone (Aldo) and angiotensin II (ANG II) are able to induce hypertension. ANG II regulates several parameters in the system like the increase of BP, the extracellular fluid volume, hormone secretion, the stimulation of sympathetic nerve activity, damping of baroreflexes and vascular and cardiac remodeling [166,167]. The activation of its receptor subtypes like AT_1a_-, AT_1b_- and AT_2_-receptors provokes physiological alterations and is consequently a possible contact point for manipulating the system. AT_1a_-, AT_1b_-receptors influences the BP and this was the pivot in the study of Vázquez *et al*. searching for siRNA to silence the subtype receptor genes. SiAT_1_ 47 directed to nucleotides 966 to 987 could be detected as a promising sequence, which silenced the AT_1_-receptor for seven days with decrease of ANG II binding to transfected cells [168]. Xue *et al*. examined with adeno-associated virus (AAV)-siRNA the silencing effect on AT_1a_-receptor and on mineralocorticoid receptor (MR) on Aldo-induced hypertension. The intracerebroventricular injected rats depicted after three weeks a 65% reduction of AT_1_-receptor protein level and 50% lower MR protein expression [169]. Aldosterone/NaCl-induced hypertension in mice could be attenuated using siRNA targeting NOX2 or NOX4, proteins of the NADPH oxidase family [170]. Another target to prevent hypertension is the calcium-permeable transient receptor potential channel (TRPC). A dysfunction in the cytosolic calcium homeostasis with an increased calcium influx by calcium-permeable ion channels seems to be responsible for pathogenesis. TRPC3 specific siRNA provoked a reduction of TRPC3 expression and a decrease in calcium influx into monocytes using spontaneously hypertensive rats [171]. In a subsequent study Liu *et al*. localized by siTRPC3 gene silencing an overexpressed TRPC3 channel protein in aortic tissue and VSMC [172].

### 10.2. Hyperlipidemia

Hyperlipidemia is known to augment the feasibility of coming down with atherosclerosis. Several therapeutic agents impairing in lipoprotein synthesis or catabolism are currently in different clinical trial phases. Recently, the FDA approved mipomersen therapy to reduce low-density lipoprotein C (LDL-C) in familial hypercholesterolaemia. Mipomersen is a 2′-*O*-methoxyethyl phosphorothioate 20-mer ASO and targets apolipoprotein B-100 (apoB-100) for reducing apoB and total cholesterol in a dose-dependent manner [173].

Another possible target for reducing LDL-C by antisense strategy is proprotein convertase subtilisin/kexin-9 (PCSK9), which interacts in the lipoprotein catabolism and is highly expressed in the liver, kidney, and intestine [174]. PCSK9 is responsible for reduced LDL receptor (LDL-R) expression and uptake of LDL leading to a higher LDL-C level in the plasma [175]. These effects are due to the binding of PCSK9 to the LDL receptor (LDL-R) that is no longer able to transport cholesterol into hepatocytes and to bind LDL-C. Gupta *et al*. achieved with a locked nucleic acid (LNA) ASO a reduction of PCSK9 mRNA of about 60% with a long-lasting effect of more than 16 days. As a consequence, the hepatic LDL-R protein could be augmented with no hepatotoxicity in a long-term study [176]. Lowering cholesterol level is also possible with RNAi using a siRNA against PCSK9. SiPCSK9 was combined with LNPs and injected intravenously in C57BL/6 mice and Sprague-Dawley rats. PCSK9 mRNA level was reduced up to 50–70% and the plasma cholesterol concentration was decreased to 40%. Testing the silencing on transgenic mice expressing human PCSK9, the reduction of transcript levels was more than 70% with a significantly decrease of the plasma protein level [177].

Lipoprotein (a) [Lp(a)] and apolipoprotein CIII (apoC-III) could also be considered for a potential treatment of hyperlipidemia. Apolipoprotein (a) [apo(a)] is covalently linked to apoB-100 to build the mature form of Lp(a). An elevated level of Lp(a) is a risk factor for CVDs. However, the treatment of transgenic mice showing kringle IV-2 repeats expression with ASO 144367 targeting apo(a) identified a high potential for reducing elevated human Lp(a) levels and furthermore the plasma apo(a) and OxPL/apo(a) [178]. ApoC-III is responsible for the concentration of plasma triglycerides and is also associated with diabetes type 2. A low level of apoC-III implicates a low triglyceride level, a fact which helps to develop a therapy approach using ASOs. Recently, Graham *et al*. successfully identified ISIS 304801 targeting the *apoC3* gene in humans, which reduced the plasma apoC-III in dose-dependent manner and the level of triglycerides in a phase I clinical study [179].

### 10.3. Diabetes

Diabetes type 1 and 2 is linked to the development of cardiovascular diseases like atherosclerosis or peripheral vascular disease and accelerates the pathogenesis in people with diabetes in comparison to healthy ones [180]. Diabetes type 1 is often associated with hyperglycemia due to destroyed ß-cells of the pancreatic islands by an autoimmune reaction which leads to a loss of insulin and an accumulation of glucose in the blood. Fas (CD95) and its ligand FasL were in focus in cyclophosphamide-induced diabetes in a model with non-obese diabetes (NOD) mice. Development of siFas has the aim of silencing Fas (CD95) for blocking the interaction between FasL, which generally provokes apoptosis of β-cells by T-cells [181]. The intraperitoneal injection at day zero and the subsequent intravenous injection of siFas at day 6 slowed down the onset of diabetes up to 40 days, whereas mice with scrambled or naked siRNA sickened within 20 days. The siRNA-PEI complexes succeeded slowing down the pathogenesis of diabetes by suppressing the Fas expression, whereas the distribution of complexes in the pancreatic islets remains unclear [182].

Recently, Okamoto *et al*. identified the gene *KCNJ15*, a risk gene for the development of diabetes, for combating diabetes type 2. *KCNJ15* mRNA is only overexpressed in the Langerhans islets in diabetic patients, caused by high glucose concentrations (25 mM) in rat insulinoma cells (INS1). The siRNA against *KCNJ15* reduced the mRNA expression in INS1 as well as the protein level. Furthermore, the insulin release of INS1 was augmented in case of incubation with 25 mM glucose after *KCNJ15* knockdown in comparison to control siRNA. Such an effect could not be seen with 5 mM glucose. Silencing *KCNJ15* in diabetic mice, the insulin secretion could be increased in siKCNJ15 treated mice in comparison to control group with scrambled siRNA [183]. As a result *KCJN15* was identified as an inhibitory modulator for glucose-stimulated insulin secretion. Another approach for treating diabetes 2 and hyperglycemia is the knockdown of the liver-specific transcription factor TORC2, which regulates the hepatic gluconeogenesis and is overexpressed in hyperglycemic rodent models [184]. High-fat diet fed C57BL/6 mice were treated with either 3 mg/mL siTORC2 or control siRNA by intravenous injection and showed a reduction in blood glucose concentrations of about 50% when siTORC2 was applied. Additionally, the genes for both enzymes involving in gluconeogenesis, phosphoenolpyruvate carboxykinase and glucose-6-phosphatase, were knocked down reducing the glucose synthesis and provoking a higher hepatic and skeletal muscle insulin sensitivity [184].

### 10.4. Stroke and Myocardial Infarction

Atherosclerosis describes the process of progressive occlusion of arteries, leading to myocardial infarction or stroke. Blockage of blood flow leads to interruption of oxygen supply and finally death of myocardial or brain cells. Transglutaminase 2 (TG2) appears in neurons. Neurons of TG2-/-mice showed significantly lower viability after oxygen-glucose deprivation (OGD), whereas astrocyte viability was significantly greater. These results could constitute an interesting basic approach for stroke prevention [185]. Another target for reducing stroke risk by knockdown with siRNA is the enzyme Caspase-3, which plays a major role in regulation of cell death, and MMP9, which is a mediator for transmigration of inflammatory leukocytes across basement membranes. A stroke model of Caspase-3 showed a downregulated caspase pathway and a brain model of MMP9 showed decreased MMP9 expression and biological activity [186,187]. Adhesion and invasion of leucocytes via the activated endothelium contribute to stroke risk after cerebral ischemia. SiRNA knocking down the VCAM receptor on endothelial cells successfully disabled binding of leucocytes with their very late leucocyte antigen-4 (VLA-4) and thus this could be another promising strategy for stroke treatment [188].

Maulik *et al*. discovered that silencing of CCR2, the chemokine receptor regulating inflammatory Ly-6Chigh monocyte subset traffic, improves recovering of apolipoprotein E–deficient (apoE−/−) mice after myocardial infarction [189]. Sugano *et al*. showed that knockdown of Src homology domain 2 (SH2) -containing tyrosine phosphatase-1 (SHP-1) reduces myocardial infarction of rats through less apoptosis and increased activation of Akt [190]. These listed examples can be extended. In conclusion there are several promising starting points for the prevention and treatment of diseases like stroke and myocardial infarction resulting from atherosclerosis.

## 11. Conclusions

In the highly industrialized countries CVDs are the leading cause of morbidity and mortality. At present, siRNA-based therapeutics, as well as ASOs, represent a new therapeutic option to treat CVDs. Due to the fact that RNAi technology has shown a fast development from the research level to human clinical trials as an effective gene-silencing method since its initial discovery in 1998, it shows great advantages for use in routine clinical practice, at least as an adjuvant of existing therapies. The challenging hurdle remains the delivery of nucleic acids *in vivo*, including the improvement of biological properties such as delivery efficacy, cellular uptake and well pharmacokinetics to achieve drug release to the desired target cell type. Nevertheless, great efforts have been already made in the establishment of siRNA delivery methods like liposomal siRNA delivery, cationic polymer-mediated siRNA delivery, targeted siRNA delivery and conjugation or chemical modifications of siRNAs. Thus, ongoing research and practical use will improve the safety issues in general, making RNAi-based therapeutics a new class of drugs with high potential, especially in the treatment of CVD.
